# Supplementary material for: Development of an IPM Strategy for Thrips and Tomato spotted wilt virus in Processing Tomatoes in the Central Valley of California
Source: Pathogens. 2020 Aug 5;9(8):636. doi: 10.3390/pathogens9080636 (PMC7459483; doi:10.3390/pathogens9080636)
Supplement: Supplementary file 1 [file pathogens-09-00636-s001.pdf]

pathogens

MDPI

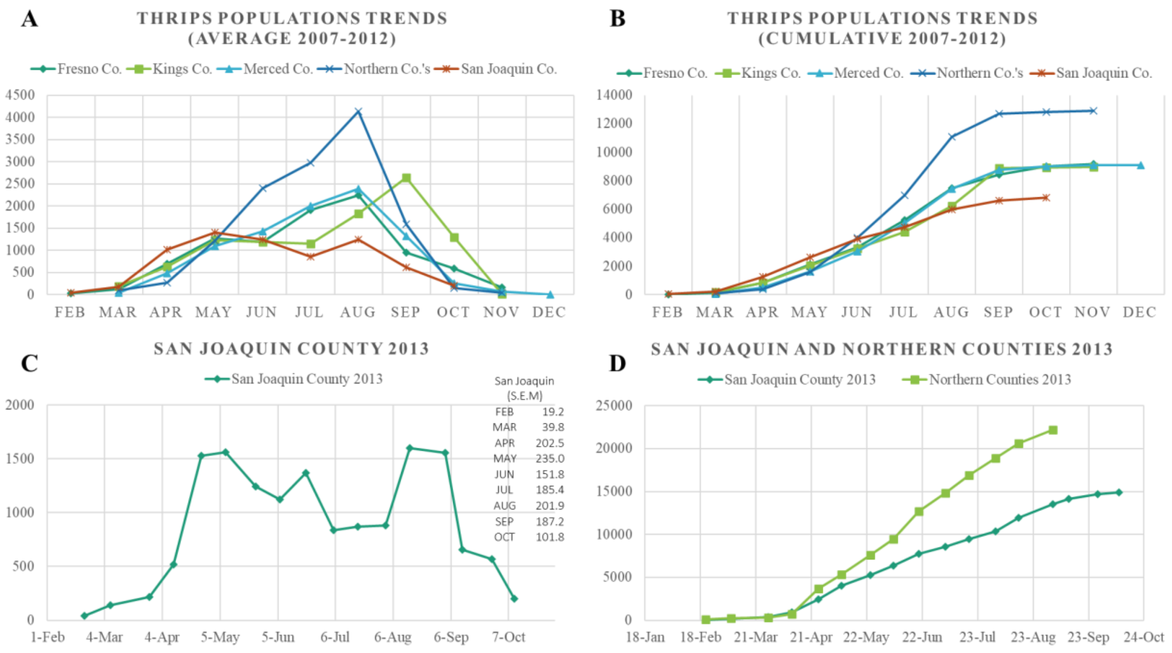

Supplemental Figure S1. Thrips population trends determined from average **(A)** and cumulative **(B)** counts made on yellow sticky cards in processing tomato fields monitored in Fresno, Kings, Merced, San Joaquin and northern counties in 2007-2012. (**C**), average thrips populations determined from counts made on yellow sticky cards in processing tomato fields in San Joaquin County in 2013. Standard error of the mean (S.E.M) is indicated in the inserted table. (**D**), Thrips population trends determined from cumulative counts made on yellow sticky cards in processing tomato fields monitored in San Joaquin and northern counties in 2013.

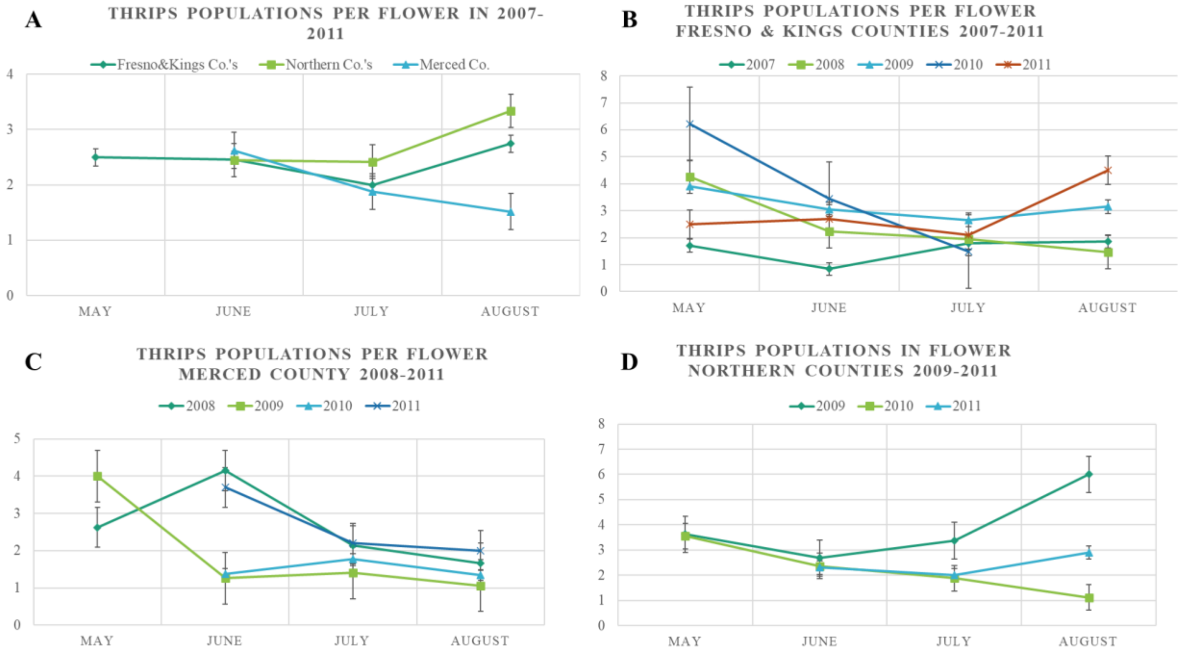

Supplemental Figure S2. Average thrips populations determined from tomato flowers in monitored processing tomato fields in 2007-2011 (numbers on the Y axis represent number of thrips/flower) (**A**), Fresno and Kings counties 2007-2011 (**B**), Merced County 2008-2011 (**C**), and northern counties 2009-2011 (**D**).

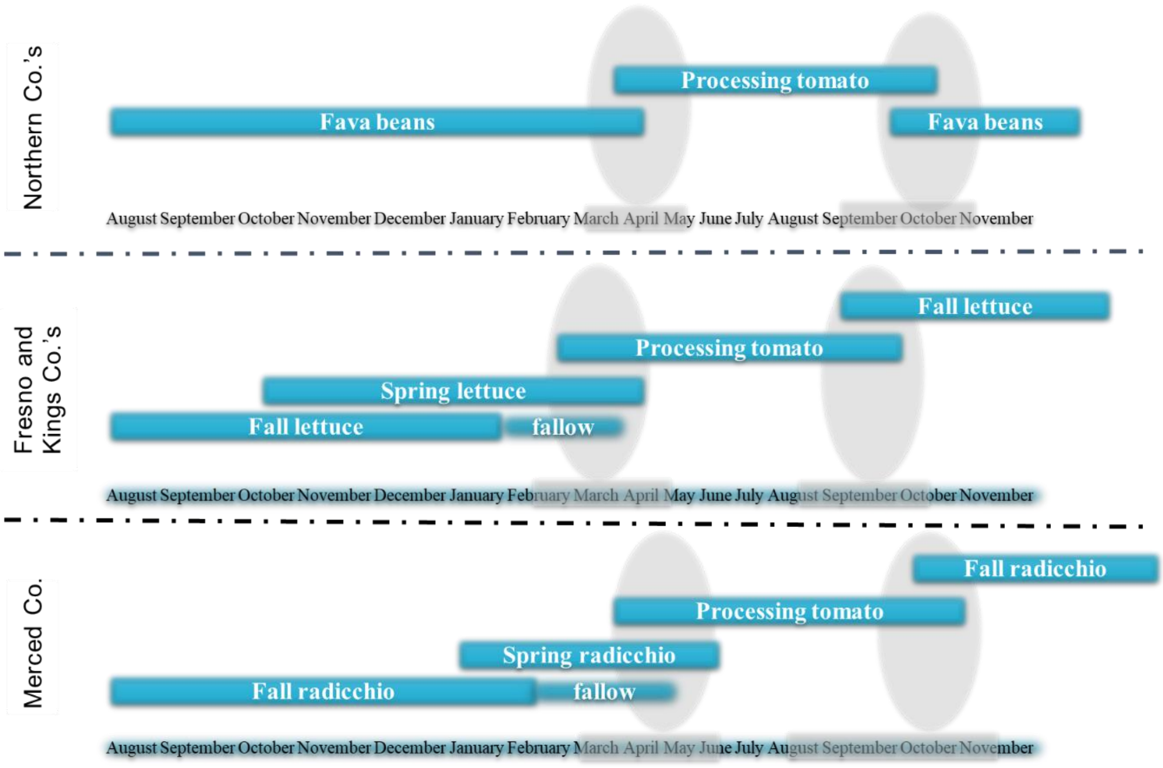

Supplemental Figure S3. Schematic presentation of how *Tomato spotted wilt virus* (TSWV) bridge crops overlap with processing tomato production in the Central Valley of California. Highlighted areas represent potential times during the year when introduction of thrips/TSWV in processing tomatoes can occur. In some cases, fall-planted lettuce and radicchio fields, left in fallow, overlapped with processing tomato fields in the spring.

Supplemental Table 1. List of fields monitored for thrips and/or *Tomato spotted wilt virus* (TSWV) in the Central Valley of California from 2007-2013.

Year	2007	2008	2009	2010	2011	2012	2013
Fresno and Kings Counties							
	7 P. Tomato	10 P. Tomato	12 P. Tomato	11 P. Tomato	10 P. Tomato	11 P. Tomato	
	1 Pepper	5 Almond	5 Almond	4 Almond	5 Lettuce	6 Wheat	
	1 Radicchio	3 Lettuce	4 Lettuce	4 Lettuce	4 Onion	4 Lettuce	
		1 FM. Tomato	3 Wheat	3 Wheat	3 Wheat	3 Radicchio	
		1 Radicchio	2 Radicchio	1 Radicchio	1 FM. Tomato	2 Onion	
			2 (Spinach and Pea)		1 Radicchio		
	**Total: 9**	**Total: 20**	**Total: 28**	**Total: 23**	**Total: 24**	**Total: 26**	
Merced County							
		4 FM. Tomato	7 Radicchio	6 Radicchio	5 P. Tomato	5 P. Tomato	
		4 Radicchio	3 P. Tomato	5 P. Tomato	3 Radicchio	2 FM. Tomato	
		3 P. Tomato	3 FM. Tomato	1 Pepper	2 FM. Tomato		
		**Total: 11**	**Total: 13**	**Total: 12**	**Total: 10**	**Total: 7**	
Northern Counties							
			9 P. Tomato	6 P. Tomato	7 P. Tomato F.	6 P. Tomato	6 P. Tomato
			1 Fava Bean	2 Fava Bean	1 Fava Bean	2 Fava Bean	2 Fava Bean
				1 Radicchio			
			**Total: 10**	**Total: 9**	**Total: 8**	**Total: 8**	**Total: 8**
San Joaquin County							
							5 P. Tomato
							**Total: 5**
**Total: 231 fields monitored (133 tomato and 98 other crops)**							

Abbreviations: FM. = fresh market; and P. = processing

Supplemental Table 2. The list of *Tomato spotted wilt virus* (TSWV) isolates used for phylogenetic tree construction.

Taxa Name	Isolate	Location	Host	Year	Gen Bank Acc. No
BR	Br20	Brazil	*Capsicum annum*	2006	DQ915948
BU -1	BS97	Bulgaria	*Nicotiana rustica*	2001	AJ418777
BU -2	10HK96	Bulgaria	*Nicotiana rustica*	2001	AJ418778
BU -3	DH37	Bulgaria	*Solanum lycopersicum*	2001	AJ418779
BU -4	GD98	Bulgaria	*Nicotiana tabacum*	2001	AJ418780
CA-1	CA-1	California, USA	*Aster sp.*	2004	AY744468
CA-2	CA-2	California, USA	*Ranunculus sp.*	2004	AY744469
CA-3	CA-3	California, USA	*Chrysanthemum sp.*	2004	AY744470
CA-4	CA-4	California, USA	*Chrysanthemum sp.*	2004	AY744471
CA-5	CA-5	California, USA	*Chrysanthemum sp.*	2004	AY744472
CA-6	CA-6	California, USA	*Chrysanthemum sp.*	2004	AY744473
CA-7	CA-7	California, USA	*Dahlia sp.*	2004	AY744474
CO	CO	Colorado, USA	*Solanum pseudolulo*	2004	AY744475
Colusa Bc1	Colusa Buttercup 13	The Central Valley of California, USA	*Ranunculus muricatus*	2013	This study
Colusa To	Colusa	The Central Valley of California, USA	*Solanum lycopersicum*	2012	This study
Colusa To1	Colusa TP To2 11	The Central Valley of California, USA	*Solanum lycopersicum*	2011	This study
Colusa To10	Colusa Esparto To 11	The Central Valley of California, USA	*Solanum lycopersicum*	2011	This study
Colusa To11	Colusa Ao To 11	The Central Valley of California, USA	*Solanum lycopersicum*	2011	This study
Colusa To2	Colusa ToWil 09	The Central Valley of California, USA	*Solanum lycopersicum*	2009	This study
Colusa To3	Colusa ToGrm 09	The Central Valley of California, USA	*Solanum lycopersicum*	2009	This study
Colusa To4	Colusa ToBD 10	The Central Valley of California, USA	*Solanum lycopersicum*	2010	This study
Colusa To5	Colusa To2TP2 09	The Central Valley of California, USA	*Solanum lycopersicum*	2009	This study
Colusa To6	Colusa To2TP1 09	The Central Valley of California, USA	*Solanum lycopersicum*	2009	This study
Colusa To7	Colusa To1TP2 09	The Central Valley of California, USA	*Solanum lycopersicum*	2009	This study
Colusa To8	Colusa To1TP1 09	The Central Valley of California, USA	*Solanum lycopersicum*	2009	This study
Colusa To9	Colusa To1BD 09	The Central Valley of California, USA	*Solanum lycopersicum*	2009	This study
Fresno Bw1	Fresno MoGlryTQ 09	The Central Valley of California, USA	*Convolvulus sp.*	2009	This study
Fresno Lt1	Fresno Let2 10	The Central Valley of California, USA	*Lactuca sativa*	2010	This study
Fresno Lt2	Fresno Let1 10	The Central Valley of California, USA	*Lactuca sativa*	2010	This study
Fresno Ns1	Fresno Nshd 10	The Central Valley of California, USA	*Solanum americanum*	2010	This study
Fresno Ns2	Fresno NightShade 11	The Central Valley of California, USA	*Solanum americanum*	2011	This study

Fresno St1	Fresno SThisFB 09	The Central Valley of California, USA	*Sonchus oleraceus*	2009	This study
Fresno St2	Fresno SThis1 09	The Central Valley of California, USA	*Sonchus oleraceus*	2009	This study
Fresno St3	Fresno SThis1 09	The Central Valley of California, USA	*Sonchus oleraceus*	2010	This study
Fresno To	Fresno	The Central Valley of California, USA	*Solanum lycopersicum*	2012	This study
Fresno To1	Fresno WSREC ToSW5 11	The Central Valley of California, USA	*Solanum lycopersicum*	2011	This study
Fresno To10	Fresno To4 10	The Central Valley of California, USA	*Solanum lycopersicum*	2010	This study
Fresno To11	Fresno To3 10	The Central Valley of California, USA	*Solanum lycopersicum*	2010	This study
Fresno To12	Fresno To3 07	The Central Valley of California, USA	*Solanum lycopersicum*	2007	This study
Fresno To13	Fresno To2 10	The Central Valley of California, USA	*Solanum lycopersicum*	2010	This study
Fresno To14	Fresno To2 08	The Central Valley of California, USA	*Solanum lycopersicum*	2008	This study
Fresno To15	Fresno To2 07	The Central Valley of California, USA	*Solanum lycopersicum*	2007	This study
Fresno To16	Fresno To1 10	The Central Valley of California, USA	*Solanum lycopersicum*	2010	This study
Fresno To17	Fresno To1 08	The Central Valley of California, USA	*Solanum lycopersicum*	2008	This study
Fresno To18	Fresno To 07	The Central Valley of California, USA	*Solanum lycopersicum*	2007	This study
Fresno To19	Fresno Lassen To1 09	The Central Valley of California, USA	*Solanum lycopersicum*	2009	This study
Fresno To2	Fresno WSREC To 11	The Central Valley of California, USA	*Solanum lycopersicum*	2011	This study
Fresno To3	Fresno ToTQ 09	The Central Valley of California, USA	*Solanum lycopersicum*	2009	This study
Fresno To4	Fresno Tosw5b 10	The Central Valley of California, USA	*Solanum lycopersicum*	2010	This study
Fresno To5	Fresno Tosw5a 10	The Central Valley of California, USA	*Solanum lycopersicum*	2010	This study
Fresno To6	Fresno ToFSds 10	The Central Valley of California, USA	*Solanum lycopersicum*	2010	This study
Fresno To7	Fresno ToFB 10	The Central Valley of California, USA	*Solanum lycopersicum*	2010	This study
Fresno To8	Fresno ToChnCrk 10	The Central Valley of California, USA	*Solanum lycopersicum*	2010	This study
Fresno To9	Fresno To5 10	The Central Valley of California, USA	*Solanum lycopersicum*	2010	This study
GA-1	WC	Georgia, USA	*Nicotiana tabacum*	1998	AF064474
GA-2	TC	Georgia, USA	*Nicotiana tabacum*	1998	AF064473
GA-3	MC	Georgia, USA	*Nicotiana tabacum*	1998	AF064472
GA-9		Georgia, USA	*Solanum lycopersicum*	1998	AF048714
GE	LE98/527	Germany	*Lysimachia sp.*	2001	AJ418781
HA-1	L	Hawaii, USA			X61799
INSV	INSV N SJC-To 2013	The Central Valley of California, USA	*Solanum lycopersicum*	2013	This study
IT	T304	Italy		1994	Z36882
JA-1	Tospo-G	Japan		2000	AB038342
JA-2	Tospo-C	Japan		2000	AB038341
JA-3		Japan		1998	AB010997

Kings Lt1	Kings Huron Let2 08	The Central Valley of California, USA	*Lactuca sativa*	2008	This study
Kings Lt2	Kings Huron Let1 08	The Central Valley of California, USA	*Lactuca sativa*	2008	This study
Kings Rd1	Kings Huron Rad5 07	The Central Valley of California, USA	*Cichorium intybus*	2007	This study
Kings Rd2	Kings Huron Rad4 07	The Central Valley of California, USA	*Cichorium intybus*	2007	This study
Kings Rd3	Kings Huron Rad3 07	The Central Valley of California, USA	*Cichorium intybus*	2007	This study
Kings Rd4	Kings Huron Rad2 07	The Central Valley of California, USA	*Cichorium intybus*	2007	This study
Kings Rd5	Kings Huron Rad1 07	The Central Valley of California, USA	*Cichorium intybus*	2007	This study
Kings To	Kings	The Central Valley of California, USA	*Solanum lycopersicum*	2012	This study
Kings To1	Kings Jayne To4 07	The Central Valley of California, USA	*Solanum lycopersicum*	2007	This study
Kings To2	Kings Jayne To3 07	The Central Valley of California, USA	*Solanum lycopersicum*	2007	This study
Kings To3	Kings Jayne To2 07	The Central Valley of California, USA	*Solanum lycopersicum*	2007	This study
Kings To4	Kings Jayne To1 07	The Central Valley of California, USA	*Solanum lycopersicum*	2007	This study
Merced Ar1	Merced Aru1 08	The Central Valley of California, USA	*Eruca sativa*	2008	This study
Merced Bw1	Merced Los Banos Morning Glory 11	The Central Valley of California, USA	*Convolvulus sp.*	2011	This study
Merced Cr1	Merced Krdn2 10	The Central Valley of California, USA	*Cynara carduncul*us	2010	This study
Merced Cr2	Merced Krdn1 10	The Central Valley of California, USA	*Cynara cardunculus*	2010	This study
Merced Cr3	Merced Kar1 08	The Central Valley of California, USA	*Cynara cardunculus*	2008	This study
Merced Gc1	Merced Groundcherry 09	The Central Valley of California, USA	*Physalis acutifolia*	2009	This study
Merced Gc2	Merced GChry 09	The Central Valley of California, USA	*Physalis acutifolia*	2009	This study
Merced Lt3	Merced Let2 07	The Central Valley of California, USA	*Lactuca sativa*	2007	This study
Merced Lt4	Merced Let1 07	The Central Valley of California, USA	*Lactuca sativa*	2007	This study
Merced Ma1	Merced Mlv 08	The Central Valley of California, USA	*Malva neglecta*	2008	This study
Merced Pe1	Merced Pep3 10	The Central Valley of California, USA	*Capsicum annum*	2010	This study
Merced Pe2	Merced Pep2BR 10	The Central Valley of California, USA	*Capsicum annum*	2010	This study
Merced Pe3	Merced Pep1BR 10	The Central Valley of California, USA	*Capsicum annum*	2010	This study
Merced Pl1	Merced PirLet2 08	The Central Valley of California, USA	*Lactuca serriola*	2008	This study
Merced Pl2	Merced PirLet1 08	The Central Valley of California, USA	*Lactuca serriola*	2008	This study
Merced Pw1	Merced PAWeed 08	The Central Valley of California, USA	*Matricaria discoidea*	2008	This study
Merced Pw2	Merced FR TSWV LG-winter pineapple weed 2011	The Central Valley of California, USA	*Cichorium intybus*	2011	This study
Merced Rd1	Merced Rad4 07	The Central Valley of California, USA	*Cichorium intybus*	2007	This study
Merced Rd2	Merced Rad3 07	The Central Valley of California, USA	*Cichorium intybus*	2007	This study
Merced Rd3	Merced Rad2 07	The Central Valley of California, USA	*Cichorium intybus*	2007	This study
Merced Rd4	Merced Rad1 10	The Central Valley of California, USA	*Cichorium intybus*	2010	This study

Merced Rd5	Merced Rad1 08	The Central Valley of California, USA	*Cichorium intybus*	2008	This study
Merced Rd6	Merced Rad1 07	The Central Valley of California, USA	*Cichorium intybus*	2007	This study
Merced Rd7	Merced FR TSWV LG-winter Radicchio 2011	The Central Valley of California, USA	*Cichorium intybus*	2011	This study
Merced Rd8	Merced FR TSWV CD-winter Radicchio 2011	The Central Valley of California, USA	*Cichorium intybus*	2011	This study
Merced Th1	Merced Thrips 07	The Central Valley of California, USA	*Cichorium intybus*	2007	This study
Merced To	Merced	The Central Valley of California, USA	*Solanum lycopersicum*	2012	This study
Merced To1	Merced (2)	The Central Valley of California, USA	*Solanum lycopersicum*		This study No
Merced To10	Merced To1 10	The Central Valley of California, USA	*Solanum lycopersicum*	2010	This study
Merced To11	Merced Patterson To 11	The Central Valley of California, USA	*Solanum lycopersicum*	2011	This study
Merced To12	Merced Newman To3 07	The Central Valley of California, USA	*Solanum lycopersicum*	2007	This study
Merced To13	Merced Newman To2 07	The Central Valley of California, USA	*Solanum lycopersicum*	2007	This study
Merced To14	Merced Newman To1 07	The Central Valley of California, USA	*Solanum lycopersicum*	2007	This study
Merced To15	Merced Gustine HT-To 11	The Central Valley of California, USA	*Solanum lycopersicum*	2011	This study
Merced To16	Merced BU3 To 11	The Central Valley of California, USA	*Solanum lycopersicum*	2011	This study
Merced To17	Merced BU2 To 11	The Central Valley of California, USA	*Solanum lycopersicum*	2011	This study
Merced To18	Merced BU To 11	The Central Valley of California, USA	*Solanum lycopersicum*	2011	This study
Merced To2	Merced Tosw5 10	The Central Valley of California, USA	*Solanum lycopersicum*	2010	This study
Merced To3	Merced ToSVR 08	The Central Valley of California, USA	*Solanum lycopersicum*	2008	This study
Merced To4	Merced ToLG1 08	The Central Valley of California, USA	*Solanum lycopersicum*	2008	This study
Merced To5	Merced ToGT1 08	The Central Valley of California, USA	*Solanum lycopersicum*	2008	This study
Merced To6	Merced ToCD3 08	The Central Valley of California, USA	*Solanum lycopersicum*	2008	This study
Merced To7	Merced To3 07	The Central Valley of California, USA	*Solanum lycopersicum*	2007	This study
Merced To8	Merced To2 07	The Central Valley of California, USA	*Solanum lycopersicum*	2007	This study
Merced To9	Merced To1MN 09	The Central Valley of California, USA	*Solanum lycopersicum*	2009	This study
Mexico To1	Mexico ToL2 09	Baja California, Mexico	*Solanum lycopersicum*	2009	This study
Mexico To2	Mexico ToF2 09	Baja California, Mexico	*Solanum lycopersicum*	2009	This study
Mexico To3	Mexico ToF1 09	Baja California, Mexico	*Solanum lycopersicum*	2009	This study
Monterey Ce1	Monterey Cel2 08	The Central Coast of California, USA	*Apium graveolens*	2008	This study
Monterey Ce2	Monterey Cel1 08	The Central Coast of California, USA	*Apium graveolens*	2008	This study
Monterey Lt1	Monterey LetRom 07	The Central Coast of California, USA	*Lactuca sativa*	2007	This study
Monterey Lt2	Monterey LetMx2 08	The Central Coast of California, USA	*Lactuca sativa*	2008	This study
Monterey Lt3	Monterey LetMx1 08	The Central Coast of California, USA	*Lactuca sativa*	2008	This study
Monterey Lt4	Monterey Let1 07	The Central Coast of California, USA	*Lactuca sativa*	2007	This study

Monterey Pe1	Monterey Pep1 08	The Central Coast of California, USA	*Capsicum annum*	2008	This study
Monterey Rd1	Monterey RadMx1 08	The Central Coast of California, USA	*Cichorium intybus*	2008	This study
Monterey Rd2	Monterey Rad2 07	The Central Coast of California, USA	*Cichorium intybus*	2007	This study
Monterey Rd3	Monterey Rad1 07	The Central Coast of California, USA	*Cichorium intybus*	2007	This study
Monterey Sp1	Monterey Spn2 07	The Central Coast of California, USA	*Spinacia oleracea*	2007	This study
Monterey Sp2	Monterey Spn1 07	The Central Coast of California, USA	*Spinacia oleracea*	2007	This study
NC -1	NC -1	North Carolina, USA	*Dahlia sp.*	2004	AY744476
NC -3	NC -3	North Carolina, USA	*Dahlia sp.*	2005	AY744478
NE	TSWV-D	Netherland		1997	AF020660
PVY CP	DD103A_184_191	South Africa	*Solanum tuberosum*		GQ853603
SA	98/0472	South Africa		2000	AJ296600
SJC Bc1	SJC Buttercup JT 13	The Central Valley of California, USA	*Ranunculus muricatus*	2013	This study
SJC Bc2	SJC Buttercup CP 13	The Central Valley of California, USA	*Ranunculus muricatus*	2013	This study
SK	KDRP	South Korea	*Capsicum annum*	2006	EF195230
SP-1	SPAIN-1	Spain	*Solanum lycopersicum*	2004	AY744479
SP-2		Spain	*Solanum lycopersicum*	2004	AY744480
SP-3	LC	Spain		1995	X94550
Yolo Bc1	Yolo Buttercup PR Bronz 13	The Central Valley of California, USA	*Ranunculus muricatus*	2013	This study
Yolo Bc2	Yolo Buttercup PR 13	The Central Valley of California, USA	*Ranunculus muricatus*	2013	This study
Yolo Em1	Davis Emilya 07	The Central Valley of California, USA	*Emilia sonchifolia*	2007	This study
Yolo Fb	Yolo Fava 09	The Central Valley of California, USA	*Vicia faba*	2009	This study
Yolo Lt1	Yolo LettuceUCD 11	The Central Valley of California, USA	*Lactuca sativa*	2011	This study
Yolo Lt2	Yolo LetCRLY 10	The Central Valley of California, USA	*Lactuca sativa*	2010	This study
Yolo Na1	Yolo Nstr2 10	The Central Valley of California, USA	*Tropaeolum majus*	2010	This study
Yolo Na2	Yolo Nast1 10	The Central Valley of California, USA	*Tropaeolum majus*	2010	This study
Yolo Pw1	Yolo PAWeed2 10	The Central Valley of California, USA	*Matricaria discoidea*	2010	This study
Yolo Pw2	Yolo PAWeed1 10	The Central Valley of California, USA	*Matricaria discoidea*	2010	This study
Yolo Pw3	Yolo PAWeed 10	The Central Valley of California, USA	*Matricaria discoidea*	2010	This study
Yolo Rd1	Yolo Rad 10	The Central Valley of California, USA	*Cichorium intybus*	2010	This study
Yolo Th1	Davis Thrips 07	The Central Valley of California, USA	*Frankliniella occidentalis*	2007	This study
Yolo To	Yolo	The Central Valley of California, USA	*Solanum lycopersicum*	2012	This study
Yolo To1	Yolo ToWint 09	The Central Valley of California, USA	*Solanum lycopersicum*	2009	This study
Yolo To10	Davis ToCA 07	The Central Valley of California, USA	*Solanum lycopersicum*	2007	This study
Yolo To11	Davis Lab2 To1 07	The Central Valley of California, USA	*Solanum lycopersicum*	2007	This study

Yolo To12	Davis Lab To1 07	The Central Valley of California, USA	*Solanum lycopersicum*	2007	This study
Yolo To2	Yolo ToMaup 09	The Central Valley of California, USA	*Solanum lycopersicum*	2009	This study
Yolo To3	Yolo ToDT1 09	The Central Valley of California, USA	*Solanum lycopersicum*	2009	This study
Yolo To4	Yolo ToDT 10	The Central Valley of California, USA	*Solanum lycopersicum*	2010	This study
Yolo To5	Yolo ToBL1 09	The Central Valley of California, USA	*Solanum lycopersicum*	2009	This study
Yolo To6	Yolo ToBDyng 10	The Central Valley of California, USA	*Solanum lycopersicum*	2010	This study
Yolo To7	Yolo To Rom 11	The Central Valley of California, USA	*Solanum lycopersicum*	2011	This study
Yolo To8	Yolo Dixon To 11	The Central Valley of California, USA	*Solanum lycopersicum*	2011	This study
Yolo To9	Yolo BD To 11	The Central Valley of California, USA	*Solanum lycopersicum*	2011	This study

BR- Brazil; BU- Bulgaria; CA-California; CO- Colorado; GE- Germany; GA- Georgia; HA- Hawaii; IT-Italy; JA- Japan; NE-Netherland; NC- North Carolina; SA- South Africa; SJC- San Joaquin County; SK- South Korea; Ar-arugula; Bc-buttercup; Bw-bindweed; Ce-celery; Cr-cardoon; Em-Emilia; Fb-fava bean; Gc-ground cherry; Lt-lettuce; Ma-Malva; Na-nasturtium; Ns-nightshade; Pe-pepper; Pl-prickly lettuce; Pw-pineapple weed; Rd-radicchio; Sp-spinach; St-sowthistle; Th-western flower thrips and To-tomato.
